# Terahertz molecular resonance of cancer DNA

**DOI:** 10.1038/srep37103

**Published:** 2016-11-15

**Authors:** Hwayeong Cheon, Hee-jin Yang, Sang-Hun Lee, Young A Kim, Joo-Hiuk Son

**Affiliations:** 1 Department of Physics, University of Seoul, Seoul 02504, Republic of Korea; 2 Department of Neurosurgery, SMG-SNU Boramae Medical Center, Seoul 07061, Republic of Korea; 3 Department of Pathology, SMG-SNU Boramae Medical Center, Seoul 07061, Republic of Korea

## Abstract

Carcinogenesis involves the chemical and structural alteration of biomolecules in cells. Aberrant methylation of DNA is a well-known carcinogenic mechanism and a common chemical modification of DNA. Terahertz waves can directly observe changes in DNA because the characteristic energies lie in the same frequency region. In addition, terahertz energy levels are not high enough to damage DNA by ionization. Here, we present terahertz molecular resonance fingerprints of DNA methylation in cancer DNA. Methylated cytidine, a nucleoside, has terahertz characteristic energies that give rise to the molecular resonance of methylation in DNA. Molecular resonance is monitored in aqueous solutions of genomic DNA from cancer cell lines using a terahertz time-domain spectroscopic technique. Resonance signals can be quantified to identify the types of cancer cells with a certain degree of DNA methylation. These measurements reveal the existence of molecular resonance fingerprints of cancer DNAs in the terahertz region, which can be utilized for the early diagnosis of cancer cells at the molecular level.

The early detection of cancer is among the most important issues in medical diagnosis because it provides a chance to treat cancer before it grows too large and spreads to other organs^1^,^2^,^3^. Many techniques from a variety of fields have been attempted to achieve early cancer detection, including optical techniques. Of these, terahertz (THz) spectroscopy shows high sensitivity for chemical changes in biological molecules without causing ionization, owing to its low photon energy^4^,^5^,^6^,^7^,^8^. THz cancer diagnostics have attracted much attention, in particular because the THz technique can distinguish normal tissue from cancer tissues via spectroscopic imaging and has been further improved with nanoparticle contrast agents to achieve higher contrast^9^,^10^,^11^,^12^,^13^. However, this method is still insufficient for cancer diagnostics because it provides only nonspecific information (location, size, and demarcation) but no molecular or biological information regarding the cancer^1^,^14^,^15^,^16^. This issue is also present in current medical imaging techniques such as magnetic resonance imaging (MRI), computed tomography (CT) and positron emission tomography (PET). These techniques require contrast agents to enhance detection and cannot provide tumour-specific information. Cancer biomarkers play a significant role in providing molecular information for the specific diagnosis of early-stage cancers^17^,^18^. In particular, carcinogenesis causes chemical and structural alterations to the DNA in cells, and detecting such changes would be important in identifying cancers. Several groups have attempted to characterize the spectral properties of DNA in the THz frequency range.

B. M. Fischer *et. al*. reported on the THz resonance features of DNA components (nucleobases and deoxynucleosides) using a THz spectroscopic system. M. Brucherseifer *et al.* measured the differences in the transmission changes and refractive indices of single- and double-stranded DNA, but no resonance peaks were found^19^,^20^. Finding spectral resonances in polynucleotide structures is difficult, although studies show that the components of DNA have many resonances in the THz range. A. G. Markelz *et al.* suggested a way to observe the chemical alteration of biomolecules in the THz region: The authors identified the collective vibration mode of protein-ligand binding (HEWL+3NAG) in a frozen solution sample in the THz range^21^,^22^. DNA methylation, an epigenetic change, is induced in genomic DNA by the chemical changes involved in carcinogenesis, such as the chemical binding of proteins. These chemical changes can be detected in polymer molecules (e.g., DNA) and in small molecules (e.g., nucleosides) in the THz region. For this reason, DNA methylation might be an effective biomarker of carcinogenesis using THz spectroscopic techniques.

Here, we show the molecular resonance fingerprints of DNA methylation in the THz region and observe them in genomic DNA from cancer cells. Furthermore, we distinguish normal DNA from cancer DNA and the type of cancer cell lines by quantifying the amplitudes of the THz resonances. Specifically, we measure the THz absorption coefficients of nucleoside samples and define THz resonance fingerprints based on the chemical changes caused by DNA methylation. We further track the THz resonances of genomic DNA samples extracted from different cell lines. The absorption coefficients of the samples are obtained using a THz time-domain spectroscopy (THz-TDS) technique that involves freezing the samples at 253 K to reduce the attenuation of the THz signal by water molecules. Finally, we quantify the degree of genomic DNA methylation based on THz absorption data and distinguish among several types of cancer cell lines using the quantified results. This study is the first to measure DNA methylation in cancer cells based on a non-labelling optical technique and will serve as a milestone for the early detection of cancer at the molecular level.

## DNA methylation in cancers

DNA methylation is an epigenetic change observed during the early stages of cancer. Although genetic changes are known to underlie carcinomatous genesis, epigenetic changes are also frequently observed in cancer tissues^23^,^24^,^25^,^26^,^27^,^28^,^29^. There are several advantages to using DNA methylation as a cancer biomarker in optical techniques. First, methylation is a universal DNA modification that can be found in most cancer types from a very early stage^30^,^31^. Second, the covalent bond formed between methyl groups and cytosine, a nucleobase, produce a very stable chemical change in the DNA^32^. THz waves are sensitive to chemical changes in molecules, and because DNA methylation is known to be among the major chemical factors influencing oncogenesis, we focus on the signals generated by these chemical changes using THz techniques. Finally, DNA methylation has the potential to serve as a universal biomarker for various types of cancer, as recent studies have reported that quantitative DNA methylation data show different trends in different types of cancer cells^28^,^33^,^34^,^35^,^36^.

**Figure 1:**
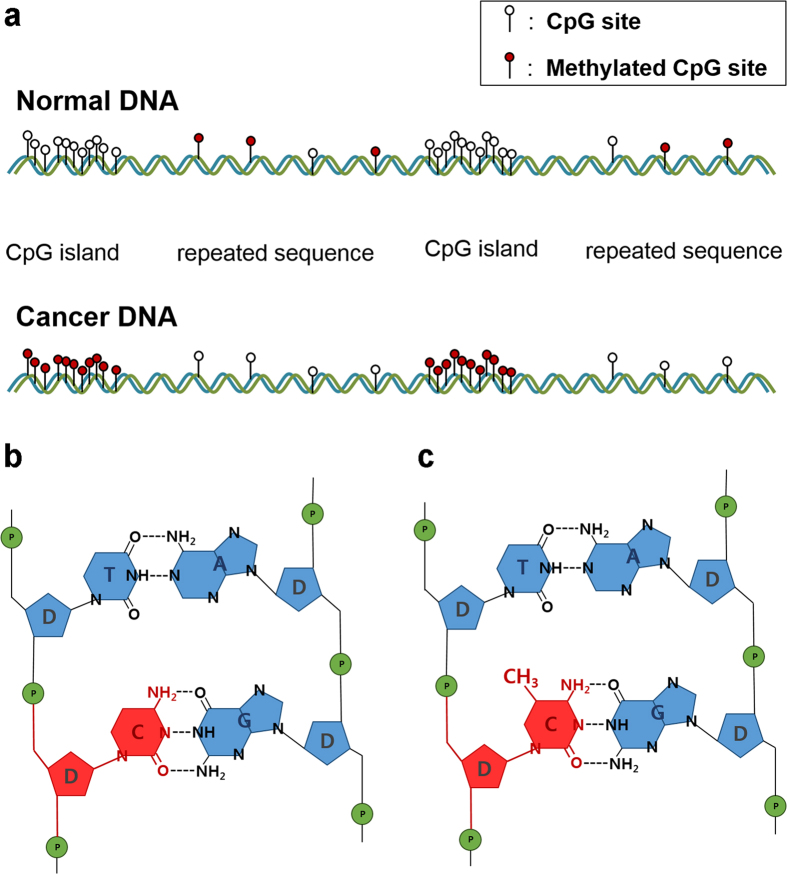
(**a**) Change in the distribution of 5-methylcytidine at CpG sites in cancer. This epigenetic change can be defined as a chemical change of the whole DNA^[37]^ and can be observed in most types of cancer. Schematic of (**b**) cytidine and (**c**) 5-methylcytidine in DNA. The conversion of (**b**) to (**c**) is called DNA methylation.

Cytosine, a nucleoside, undergoes chemical changes wherein a methyl group is attached to the fifth carbon atom in the sugar ring to generate 5-methylcytosine. Normal DNA CpG islands (CGIs: high-frequency CpG sites within a promoter, which comprise a paired cytosine and guanine) are protected from methylation, such that 5-methylcytosine is mostly found at CpG sites in repeated sequences. Conversely, in cancer DNA, hypermethylation occurs at CGIs, and hypomethylation occurs in the repeated sequences^37^,^38^ (Fig. 1a). Therefore, this change can be measured at the whole-DNA level by comparing the methylation degree of cancer and normal DNA (Fig. 1b,c).

## THz fingerprints of the chemical analogues of cytidine

**Figure 2:**
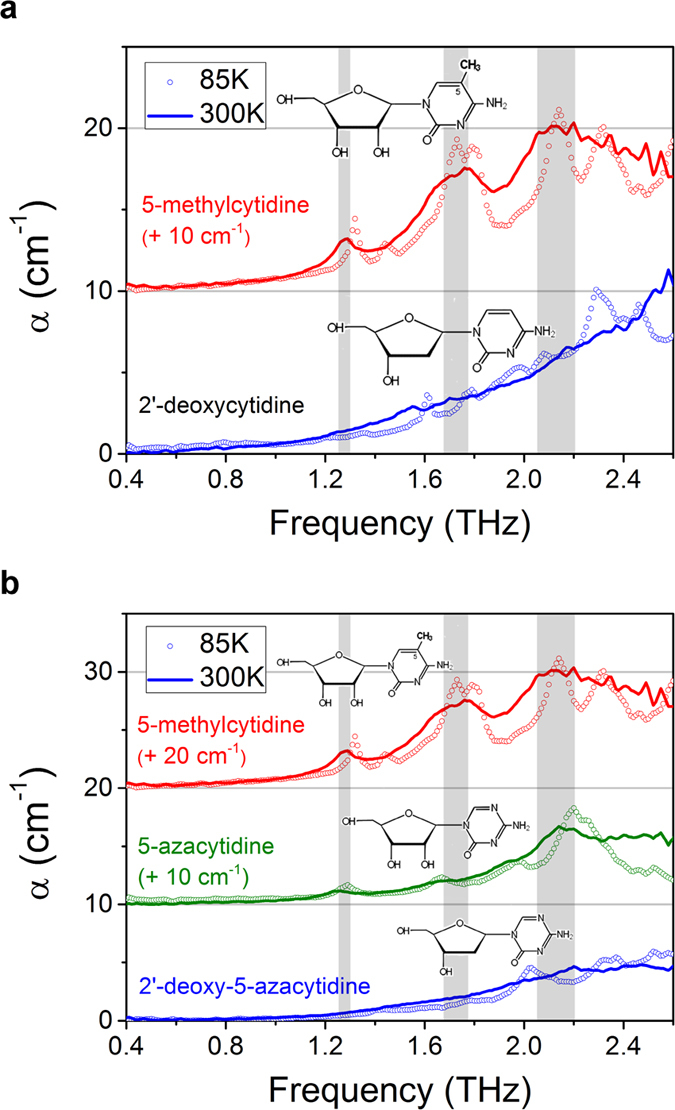
(**a**) Absorption coefficients in the 0.4–2.5 THz range for 2′-deoxycytdine and 5-methylcytidine. (**b**) Absorption coefficients of 5-azacytidine and 2′-deoxy-5-azacytidine compared with 5-methylcytidine. (inset) Chemical structures of 2′-deoxycytdine, 5-methylcytidine and two analogues of cytidine: 5-azacytidine and 2′-deoxy-5-azacytidine. 5-methylctyidine has a methyl group on the fifth carbon in cytidine, which is replaced with nitrogen in the analogues. The methyl group changes the collective vibrational mode and structure of cytidine molecules, and this change emerges as three major resonance peaks in the THz region. These three resonance peaks serve as spectral fingerprints for methylation in DNA-level experiments.

We prepared 2′-deoxycytidine (2′-dC) and 5-methylcytidine (5-mC) as unit models of nucleoside molecules that present a change in DNA cytidine levels with cancer^39^. We measured of 2′-dC and 5-mC pellets to obtain molecular resonance fingerprints for methylation at room temperature (300 K) and 85 K. Three major 5-mC absorption peaks were observed at 1.29, 1.74, and 2.14 THz at room temperature, but 2′-dC did not show any distinct peaks in the range of 0.4–2.5 THz. At a lower temperature (85 K), the 5-mC peaks were divided and sharpened; 2′-dC also showed small peaks at 85 K, but these did not coincide with the 5-mC peaks (Fig. 2a). We then investigated whether these peaks coincided with the methylation of the nucleoside. Figure 2b shows the absorption coefficients of two chemical analogues of 2′-dC: 5-azacytidine and 2′-deoxy-5-azacytidine. These molecules have a few weak resonances that differ from the 5-mC peak positions at both room temperature and 85 K. This result indicates that the three major resonance peaks of 5-mC are THz fingerprints of nucleoside methylation.

## Looking for THz molecular resonance in cancer DNAs

The molecular resonance fingerprints of methylation obtained from the THz spectral changes of the nucleosides were tracked in genomic DNA samples. Water molecules minimally influence the fifth carbon of cytidine (where the methyl group is attached) in DNA in aqueous solution^40^,^41^. We therefore assumed that the molecular resonances of methylation observed at the nucleoside level would similarly appear at the DNA level.

For the experiments at the DNA level, we prepared normal cellular DNA (293 T cells) and the corresponding methylated DNA (methylated 293 T, M-293T), in which cytidine was artificially changed to 5-mC. Furthermore, we obtained cancer DNA from five types of human cancer cell lines (PC3, a prostate cancer cell line; A431, an epidermoid carcinoma cell line; A549, an adenocarcinomic lung cell line; MCF-7, a breast cancer cell line; and SNU-1, a gastric carcinoma cell line) to identify methylation signals in cancer genomic DNA. The solution samples were frozen at 253 K to minimize attenuation by water molecules. Ice in DNA solutions has no resonance in the THz range^42^ but produces a background that is predicted to have higher absorption than methylation resonance peaks. The amplitude of the absorption peaks in the nucleoside is less than 10 cm^−1^, whereas ice exhibits absorption coefficients of a few tens cm^−1^ in the THz range^43^.

The frozen samples were attached to a quartz window and maintained at a constant temperature (253 K) during the THz-TDS measurement process. Each measured data point in each sample was averaged three times in the time domain, and all measured data were averaged three times. Time-domain data were converted into frequency-domain data using a fast Fourier transform (FFT) algorithm. We determined the absorption coefficient of the samples by comparing the sample data and the reference data (derived from a single window only). The absorption coefficient data of seven DNA samples (293 T, M-293T, PC3, A431, A549, MCF-7, and SNU-1) showed a trend of ice in the THz range but indicated bumps around 1.6 THz. The absorption coefficient of ice was measured to determine the baseline shape, given that ice has a large influence on the measured data due to its high volume in the solution sample. The final measured spectra decreased to 2.0 THz due to the strong attenuation by ice. From the shape of the ice absorption coefficient, a Gaussian distribution was chosen as the baseline function for analysing the experimental results. To remove the ice background from the results, we fitted the DNA data using the absorption coefficient of ice as the baseline.

**Figure 3:**
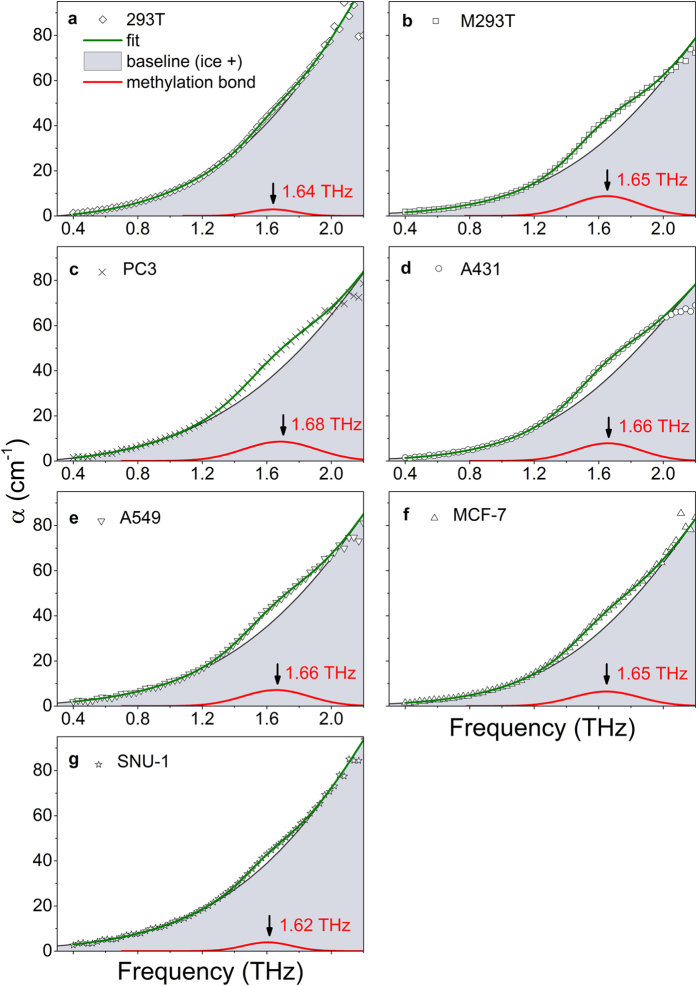
Absorption coefficients of (**a**) normal DNA, 293 T and (**b**) artificially methylated DNA, M-293T. We obtained THz absorption coefficients for DNA from cancer cell lines – (**c**) PC3; (**d**) A431; (**e**) A549; (**f**) MCF-7; (**g**) SNU-1 - using the same method. Experimental data were measured with a THz-TDS system at 253 K. The data are valid between 0.4 THz and 2.0 THz because ice heavily attenuates the THz signal at high frequency. These results show the trend of the ice absorption coefficient in the THz range but also a bump approximately 1.6 THz. The data were fitted well by a superposition of two Gaussian functions (green line). The baseline (grey line) is the first Gaussian function, representing ice and other buffer components. The red line shows another Gaussian function, which we propose to be the resonance peak of the methylation bond.

**Figure 4:**
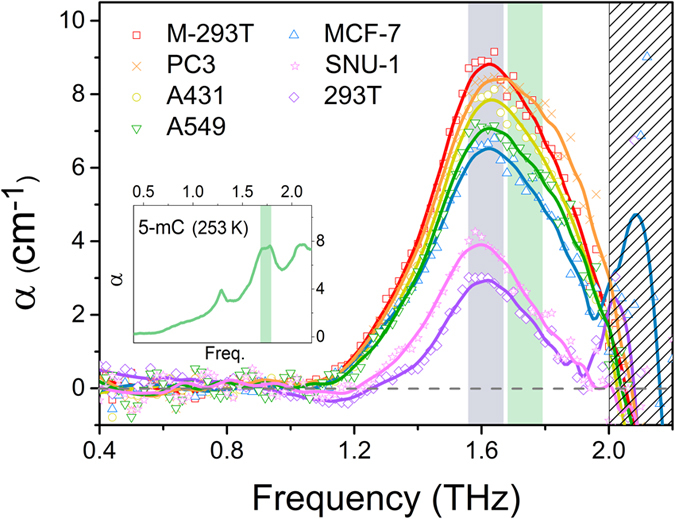
THz resonance peaks for DNA samples after baseline correction. The first Gaussian function (the absorption coefficient of ice and other buffer materials) of Fig. 3 acts as the baseline for each measured data point. The results were obtained by subtracting the baseline from the measured data at each data point. There is only one resonance peak (~1.67 THz), but its position is similar to the second peak (1.74 THz) for the 5-mC result (emerald line). We attributed the peak shift to interactions with water molecules in aqueous solvent.

The shapes of most ideal spectral resonance peaks are determined by Lorentzian, Voigt, or Gaussian functions, but inhomogeneous broadening alters these to the form of actual spectral resonance features. We fitted the measured data to find resonance peaks with such functions. All of the measured DNA sample data fit well with a superposition of two Gaussian functions within a valid measurement range of 0.4–2.0 THz (Fig. 3). One of the Gaussian functions is on one side of the edge of a large Gaussian function that can be regarded as the ice background, extra structure from DNA, and extra materials (agents added to maintain DNA structure). This baseline Gaussian function is strongly influenced by ice. Another small Gaussian peak, which causes the bump around 1.6 THz in the absorption coefficient of the measured data, represents the resonance peak of these samples, which we attribute to the DNA methylation bond. We obtained a resonance peak from each sample data point by subtracting the baseline Gaussian function from the measured data. Figure 4 shows the resonance peaks of all samples in the range of 0.4–2.0 THz acquired after baseline subtraction. The peak of the Gaussian bump for the fitting curves was approximately 1.67 THz, which is similar to the position of the absorption peak of 5-mC (1.74 THz), but with the centre of the peak shifted^44^.

## Quantification of DNA methylation

**Figure 5:**
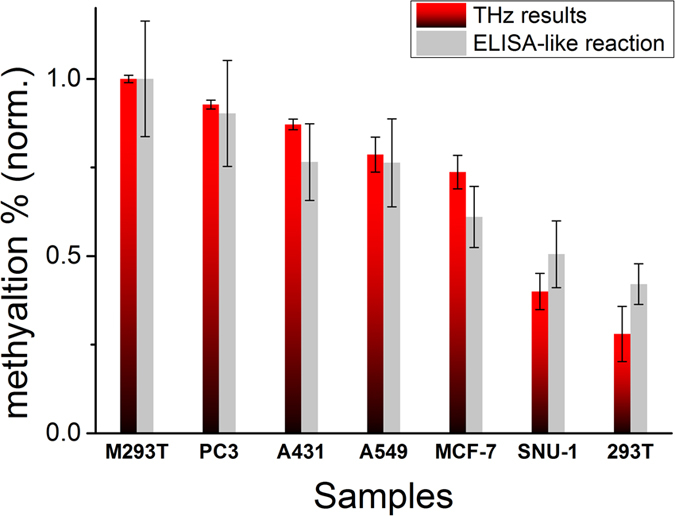
The degree of DNA methylation for each DNA sample. The results were compared with quantitative data from a commercial biological method (ELISA-like reaction). The data from both methods were normalized based on the degree of the M-293T sample, which had the highest methylation ratio. THz results were quantified by the height of the resonance peak of the baseline-corrected results and baseline values.

The amplitudes of the absorption peaks at 1.67 THz in Fig. 4 are believed to correspond to the degree of DNA methylation in the DNA samples. Therefore, if the degree of DNA methylation depends on the cancer cell type, quantifying the value of the absorption coefficient could enable classification. The absorption coefficients of the DNA samples were quantified based on the highest point values of the peaks at 1.67 THz. The quantified values were compared with a commercial DNA methylation quantification measurement (enzyme-linked immunosorbent assay like reaction method (ELISA)-like reaction method, Epigentek Group, Inc.) for verification^45^ (Fig. 5). The ELISA-like reaction data were averaged several times. The data from the two techniques cannot be compared directly because they were gathered using different measurement principles. We set 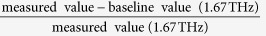
as the comparison value for the THz experiment data to make them similar to the biological method and normalized them based on M-293T. The results of these two methods showed good agreement, and we determined that the resonance peak of DNA methylation can be used to quantify the degree of DNA methylation.

## Discussion

**Figure 6:**
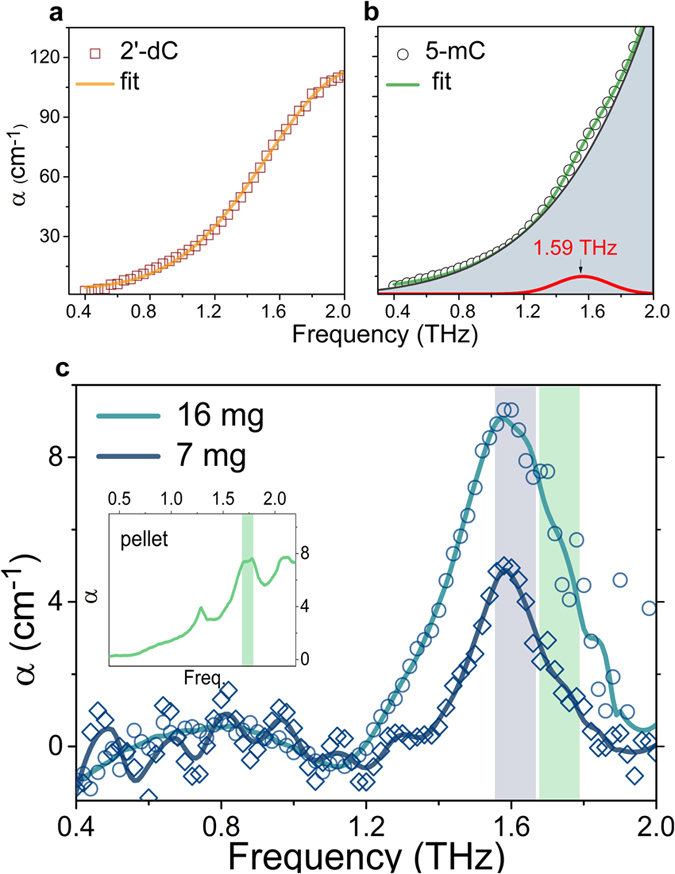
(**a**) THz absorption coefficients of 2′-deoxycytidine (2′-dC) and (**b**) 5-methylcytidine (5-mC) in aqueous solution. The solutions were made of 300 ml distilled water and the same weight of nucleoside sample. The absorption coefficients of the solutions were measured by the THz-TDS technique at 253 K. The 2′-dC solution was well fitted with a single Gaussian function, with no resonance peak, as was the case for ice. The 5-mC aqueous solution in the frozen state was fitted well with a double Gaussian function, with a small resonance peak at 1.59 THz. The resonance peak could be separated by baseline correction using the same method as for DNA solutions. (**c**) The heights of the resonance peaks were proportional to the amounts of 5-mC in the solutions. The position of the resonance peak for the 5-mC solution moved to a lower frequency than that of the 1.74-THz peak of the 5-mC pellet (inset). The red shift was also observed in the DNA sample results.

In this study, we observed the THz molecular resonance fingerprints of DNA methylation from nucleoside molecules and tracked them at the genomic DNA level. As reported in previous studies, the resonance signals of nucleosides within a DNA structure are difficult to obtain mainly due to broadening by the polymerization of mononucleotides and the inhomogeneous broadening of macromolecules. Because DNA methylation is not affected by nucleoside polymerization, we chose DNA methylation as a marker to follow the chemical changes in DNA induced by carcinogenesis. We followed a single resonance peak of DNA methylation from nucleosides to genomic DNA and examined whether the peak represented the signal of DNA methylation in the solution state. Figure 6 shows the THz absorption coefficient of aqueous solutions of 2′-dC and 5-mC at 253 K: 2′-dC did not show a significant resonance feature in this range; thus, the absorption coefficient in solution could be fitted with a single Gaussian function similar to that of ice. In contrast, the 5-mC solution data showed a resonance peak at 1.60 THz (Fig. 6b). The previous data for a pellet of 5-mC showed two major resonance peaks at 1.29 and 1.74 THz, and the 1.29 THz peak was smaller and narrower than the 1.74 THz peak. This result may indicate that the THz absorption signal of the 1.29 THz peak is too weak to be seen because of the high attenuation by ice; the 1.74 THz peak might be moved to a lower frequency because of the interaction with water molecules^45^. The intensity of the 1.60-THz peak changed as the concentration varied in solution and was quantified (Fig. 6c). The 5-mC resonance peak seemed to be reflected in the absorption coefficients of DNA solutions, and our THz-TDS technique allowed us to observe the THz molecular resonance of DNA methylation at the DNA level. These results indicate that THz spectroscopy can be used to observe epigenetic changes induced by carcinogenesis and can be a powerful method to diagnose early-stage cancer. We expect that an ultrasensitive THz sensor might be utilized to find other resonance peaks from DNA samples by amplifying the absorption cross-section of THz signals in fixed narrow-frequency regions^46^. Furthermore, the ability to observe resonance peaks at the cellular level *in vivo* via highly sensitive THz techniques could enable the development of a novel cancer imaging system^1^,^47^. This research demonstrates that DNA methylation may be among the strongest probes for chemical changes in DNA for use in early cancer detection via optical techniques^48^,^49^,^50^.

## Conclusion

We detected THz molecular resonance fingerprints caused by the methylation of cancer DNA extracted from living cell lines and quantified them to distinguish cancer types. Two major absorption peaks (1.29 THz and 1.74 THz) for methylation were identified between 0.4 THz and 2.0 THz by comparing two nucleoside samples, 2′-deoxycytidine (2′-dC) and 5-methylcytidine (5-mC), as well as chemical analogues. Additionally, we tracked the resonance features of genomic DNA solution samples (293 T DNA and artificially methylated 293 T DNA) at 253 K. A single absorption peak was observed at 1.67 THz for both DNA samples by applying a baseline correction method. This peak is located at a lower frequency than the second peak (1.74 THz) of the nucleoside result. We concluded that the peak would be moved to a lower frequency by the solution state of the samples and examined it through comparison with the results of nucleoside solutions. We also found the same resonance peaks at 1.67 THz in genomic DNA from various types of cancer (PC3, A431, A549, MCF-7 and SNU-1 cell lines). The magnitude of the resonance peak of each sample was quantified using the amount of genomic DNA methylation. The results were similar to those obtained using a commercial biomedical quantification technique. In conclusion, we discovered that the methylation signal of cancer DNA in the THz range can be used to distinguish between normal and cancer DNA.

## Methods

No statistical methods were used to predetermine the sample size. No samples were excluded from the data analysis.

### Spectroscopy system

Measurements were performed using a THz time-domain spectroscopy (THz-TDS) system based on a Ti:sapphire femtosecond oscillator (Synergy; Spectra-Physics) pumped by a 10-W diode laser, Verdi. The laser delivered 10-fs pulses at a wavelength of 800 nm with an 80-MHz repetition rate. The laser beam was separated by a polarizing beam-splitter and turned towards the emitter and the detector separately. The emitter was a p-InAs crystal that utilized the photo-Dember effect, and the laser beam was incident at 78° on the surface of the crystal for high THz intensity^51^. The THz beam that radiated from the emitter was gathered by a parabolic mirror and focused onto the sample holder by a pair of THz focusing lenses (Tsurupica; Microtech Instrument, Inc.) after being collected by a parabolic mirror (Supplementary Fig. 1a). The THz beam was focused on the centre of the gap of the detector, a 5-μm-gap, parallel-line, photoconductive antenna (PCA) (PCA-40-06-10-800-h; Batop), using a hyperhemispherical silicon lens. The THz beam was probed on PCA by another laser beam, which was separated by the beam-splitter. The spectroscopic system and the sample holder were purged with dry air (>2% humidity) and confined in a closed box to reduce the absorption of water vapour and the frost on the surface of the window of the sample holder.

### Temperature-controlled sample holder

We used a cryostat (ST-100-FTIR; Janis Reserch Company) for the pellet samples but prepared a temperature-controlled sample holder system for the liquid samples. The sample holder was specially designed to freeze aqueous solution samples because THz waves are strongly attenuated by water. Several groups have used a drying method on various windows (sapphire, silicon, or Teflon plate, for aqueous samples). However, this method produces the so-called “coffee-ring effect” (non-uniform concentrations of molecules on the plate after the solution evaporates)^52^,^53^. To reduce the attenuation by water without the coffee-ring effect, the samples were frozen during measurement. The sample holder contacted a pair of thermoelectric coolers that maintained the temperature of the sample at −20 °C (253 K) with ± 0.05 °C variation. Two quartz windows were needed to flatten the frozen sample disk, but one window was removed to reduce attenuation by the window after the sample was frozen. The removed upper window was made of a hydrophobic material (Teflon), and its surface was treated to be more hydrophobic (the contact angle was larger than 100°) (Supplementary Fig. 1b). The frozen samples were stably attached on a single quartz window during THz scanning at very low humidity.

### Method to obtain the optical index

The reference THz spectral range was 0.4–3.5 THz. Absorption coefficients were obtained by comparing the frequency-domain THz spectra derived from the FFT of the time-domain THz waveforms through the reference and a sample. The sample and reference signals were represented by Fresnel′s equation:


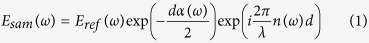


where *E*_*sam*_(*ω*) is the signal that interacts with a sample; *E*_*ref*_(*ω*) is the reference signal without sample interaction; *d* is the propagation thickness through a sample; and α(ω) and *n*_1_(ω) are the absorption coefficient and the real part of the complex refractive index of a sample, respectively. The absorption coefficient α(ω) can be expressed as:


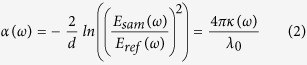


where *λ*_0_ is the wavelength of THz in vacuum, and *κ(ω*) is the imaginary part of the refractive index. These relationships are solved through the method proposed by L. Duvillaret *et al.*, which uses a numerical optimization algorithm^54^.

### Nucleoside samples

**Figure 7:**
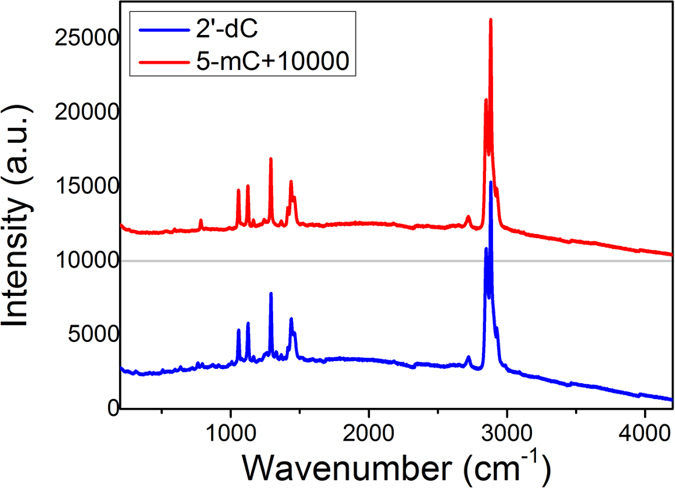
Raman resonant scattering spectra for 2′-dC (blue line) and 5-mC (red line) pellet samples from 200 to 4200 cm^−1^ (6–1300 THz). It is difficult to distinguish the differences in resonance features between the two samples, although they are discriminated in the THz region.

Nucleoside samples, which are nucleobases attached to a sugar, were prepared because nucleosides are more structurally stable than nucleobases. The samples were purchased from Sigma-Aldrich Co. and formed into pellets with a thicknesses of 1.5–2.0 mm after dilution at a 1:36 mass ratio (sample:HDPE) with high-density polyethylene powder (HDPE, Sigma-Aldrich Co.), which has very low absorption (under 4 THz) and a constant refractive index (~1.54). The powder mixture was compressed in a pellet cast at 1500 psi for 5 min to form pellet samples. The pellet samples had stable planform surfaces for transmission spectroscopy. Their THz characteristics were measured in a cryostat at room temperature (300 K) and at 85 K. To characterize the THz optical properties of nucleoside methylation, 2′-dC and 5-mC were measured and compared at the aforementioned temperatures (Fig. 3a). The resulting resonant absorption features were used to obtain the nucleoside methylation fingerprints. The results were also compared with other chemical analogues of cytidine, namely, 5-azacytidine and 5-aza-2′-deoxycytidine, in which the fifth carbon is replaced by nitrogen (Fig. 3b). We also measured the spectral signal of nucleoside samples using another optical technique, Raman spectroscopy. Measurements of pure 2′-dC and 5-mC pellet samples were performed from 200 to 4200 cm^−1^ (6–1300 THz) using a triple-grating Raman spectrometer (Fluorolog-3, Horiba) driven by a 25-mW, 513-nm sapphire laser (Sapphire LP System, Coherent). The pellet samples were made from pure 80-mg 2′-dC and 5-mC powder samples with PE mixture samples because the HDPE powder shows resonance in the measured spectral range. The pellets were only a few microns thick. Figure 7 shows the Raman spectra of the pellet samples. A slight difference was observed in the small resonance peaks in the low-frequency range; however, in most regions, the major resonance peaks of the two samples were very similar, in contrast to the THz results.

### Extracted DNA samples

All samples for this study were treated under ethical approval which was obtained from institutional board in Seoul National University Boramae Medical Centre. A genomic DNA sample consisting of 2′-dC and 5-mC (Fig. 1) was prepared from 293 T cells (human embryonic kidney) using the QIAamp DNA Mini Kit (Qiagen). The 293 T DNA solution was divided in two, and one portion was artificially methylated (methylated 293 T, M-293T) using the CpG methyltransferase enzyme (New England Biolabs Ltd.) according to the manufacturer’s protocol. The concentration of each solution sample was approximately 500 ± 7 μg/ml. We investigated whether the spectral resonance features of methylation could be observed in cancer DNA, and the degree of methylation was higher than that of normal DNA. Five cell lines (PC3, a prostate cancer cell line; A431, an epidermoid carcinoma cell line; A549, a lung cancer cell line; MCF-7, a breast cancer cell line; and SNU-1, a gastric cancer cell line) were chosen as cancer DNA samples. The cancer DNA samples, models for different types of cancers, were obtained from the Korean Cell Line Bank. We treated these cells with the same method as the 293 T cells, and all DNA samples were dissolved in distilled water. The samples were dropped on a z-cut quartz window with a 300-μm copper spacer covered with an upper window and then frozen at 253 K for 5 min. The z-cut quartz window has high transmittance (approximately 80%) and a constant refractive index (~2.1), which is similar to that of ice, thereby minimizing signal loss. After the sample was completely frozen, the upper window was removed and measured while the sample holder was maintained at the same temperature.

### Separation of the DNA methylation signal from the measured data

The absorption coefficients of the DNA samples included not only the DNA signal but also the baseline signal arising from the other materials used to make the DNA samples. Because the materials included ice, which has higher absorption than the HDPE powder, baseline correction was required to find the resonance in the absorption^55^,^56^. We used Matlab 2011 and Origin Pro 9, a software package for numerical computation and visualization, to calculate the baseline and to fit the measurement data. First, we set the form of the baseline to be subtracted from the measured THz data. We measured the absorption coefficient of ice to be somewhere in the range of 0.1–2.0 THz and fitted it to determine the baseline form because we expected that the sample signal would be mostly influenced by ice, which represented the largest volume in the samples. The measured ice data were fit best by a Gaussian function in our THz window (Supplementary Fig. 2). Through baseline correction using the ice fit, we were able to separate the methylation resonance peaks from DNA measurement data.

### Quantification of the DNA methylation signal

We used a commercial biological quantification method for global DNA methylation, the Methylamp Global DNA Methylation Quantification Ultra Kit (ELISA-like reaction, Epigentek, Inc.), to validate our THz quantification technique^48^,^57^. We set the highest point value of 1.67 THz as the degree of DNA methylation. To compare the data with the results of the ELISA-like reaction technique, we defined the comparison value as


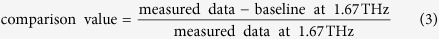


The degree of DNA methylation from the ELISA-like reaction technique is shown in Supplementary Table 1 and was calculated as


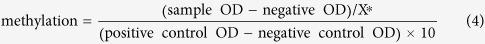


where OD is optical density, and X^*^ is CG (cytosine – guanine) content (human DNA: 41%). Finally, both quantities were normalized to the value of the M-293T sample as a reference for direct comparison.

## Additional Information

**How to cite this article**: Cheon, H. *et al.* Terahertz molecular resonance of cancer DNA. *Sci. Rep.* **6**, 37103; doi: 10.1038/srep37103 (2016).

**Publisher’s note:** Springer Nature remains neutral with regard to jurisdictional claims in published maps and institutional affiliations.

## Supplementary Material

Supplementary Information
