# Supplementary material for: Terahertz molecular resonance of cancer DNA
Source: Sci Rep. 2016 Nov 15;6:37103. doi: 10.1038/srep37103 (PMC5109182; doi:10.1038/srep37103)
Supplement: Supplementary Information [file srep37103-s1.pdf]

Terahertz molecular resonance of cancer DNA

Hwayeong Cheon[1], Hee-jin Yang[2], Sang-Hun Lee[1], Young A Kim[3] and Joo-Hiuk Son[1†]

[1] Department of Physics, University of Seoul, Seoul 02504, Republic of Korea

[2] Department of Neurosurgery, SMG-SNU Boramae Medical Center, Seoul 07061, Republic of Korea

[3] Department of Pathology, SMG-SNU Boramae Medical Center, Seoul 07061, Republic of Korea

[†] To whom correspondence should be addressed: joohiuk@uos.ac.kr

Keywords: terahertz, DNA methylation, molecular resonance, optical diagnosis of cancer

Supplementary information

Measurement system.

The THz time-domain spectroscopy (THz-TDS) system consisted of a Ti:sapphire femtosecond oscillator (Synergy; Spectra-Physics) pumped by a 10-W Verdi diode laser. The 10-fs pulse laser was used at a wavelength of 800 nm, with an 80-MHz repetition rate. A polarizing beam-splitter was used to separate the laser beam, and the separated beams were sent towards the emitter and the detector. The emitter was a p-InAs crystal that utilized the photo-Dember effect, and the laser beam was incident at 78° on the surface of the crystal for high THz intensity. The emitting THz beam was gathered by a parabolic mirror and focused onto the sample holder by a pair of THz focusing lenses (Tsurupica; Microtech Instrument, Inc.), which have high transmission and low surface loss for THz waves. The THz beam was focused on the centre of the gap of the detector, a 5-μm-gap, parallel-line, photoconductive antenna (PCA) (PCA-40-06-10-800-h; Batop), using a parabolic mirror and a hyperhemispherical silicon lens. The THz beam was probed on PCA using a laser beam on the detector. The spectroscopic system and the sample holder were confined in a closed box under 2% humidity to reduce the absorption of water vapour and prevent the surface of the window of the low-temperature sample holder from frosting over. The pellet samples were measured by a cryostat (ST-100-FTIR; Janis Research Company), and the liquid samples were measured on a temperature-controlled sample holder. The sample holder was specially designed to freeze aqueous solution. The sample holder was contacted a pair of thermoelectric coolers that maintained the temperature of the sample at -20 ℃ (253 K) with ± 0.05 ℃ variation. Two z-cut quartz windows were needed to flatten the frozen sample disk. The upper window, which removed after the freezing process, was made of a hydrophobic material (Teflon), and its

surface was treated to be more hydrophobic (the contact angle was larger than 100°). The frozen samples were stably attached on a single quartz window during THz scanning at very low humidity.

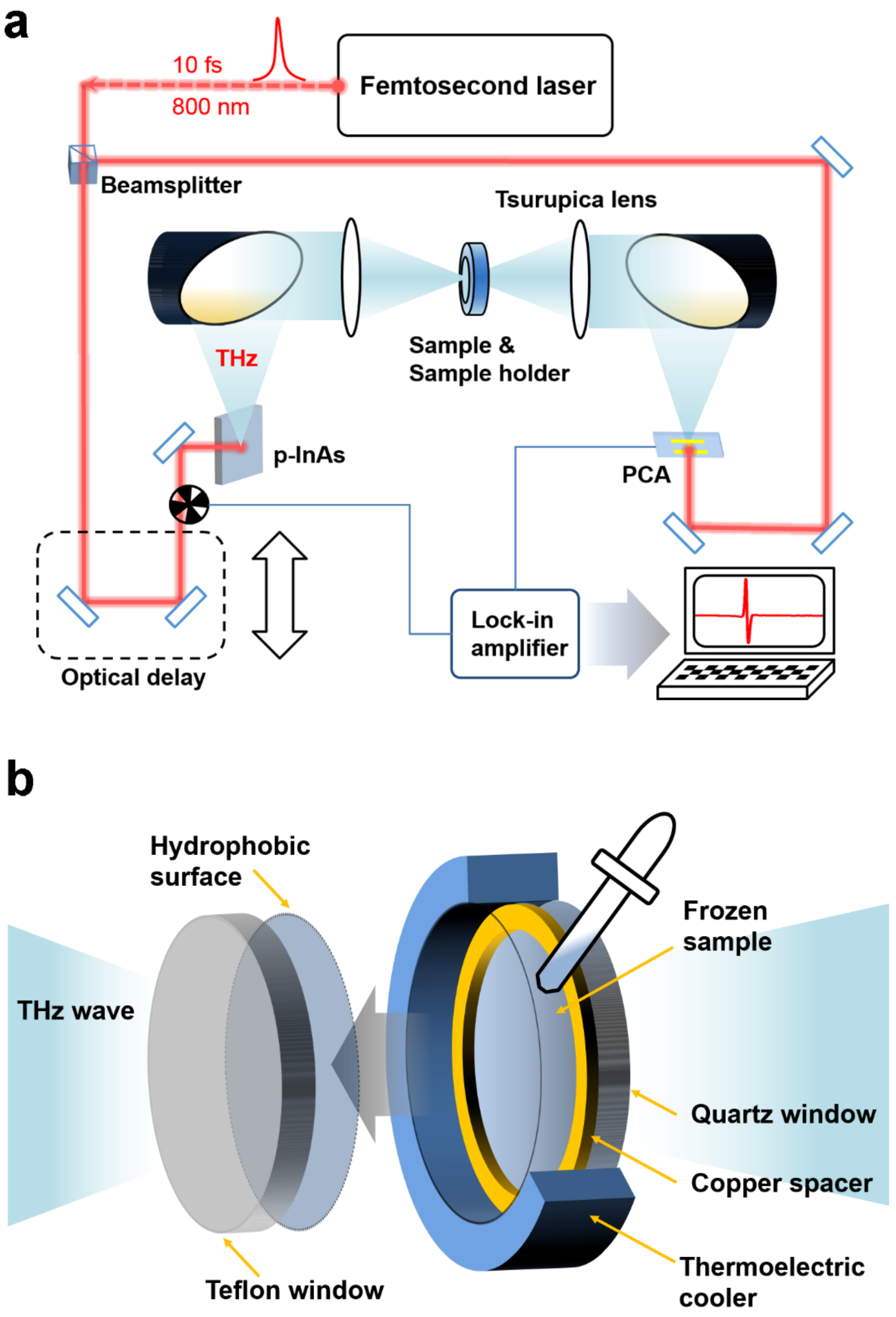

Supplementary Figure 1 | (**a**) THz-TDS system and (**b**) the schematic of a temperature-controlled sample holder. The THz beam was focused onto the centre of the sample holder and transmitted through the sample and a single quartz window. A Teflon window flattened the surface of the frozen liquid samples to calculate the optical coefficients using Fresnel's equation, and was removed before the measurement to reduce attenuation by the window[52]. We measured the THz waveforms of the samples after the temperature stabilized at 253 K.

Trend curves of absorption coefficient of ice.

The absorption coefficient of ice was measured to be somewhere in the valid range from 0.1–2.0 THz and fitted to determine the baseline form. Ice has the influence on the sample signal because ice constitutes the bulk of the sample. The measured ice data were fit best by a Gaussian function in the valid THz range (Supplementary Fig. 2).

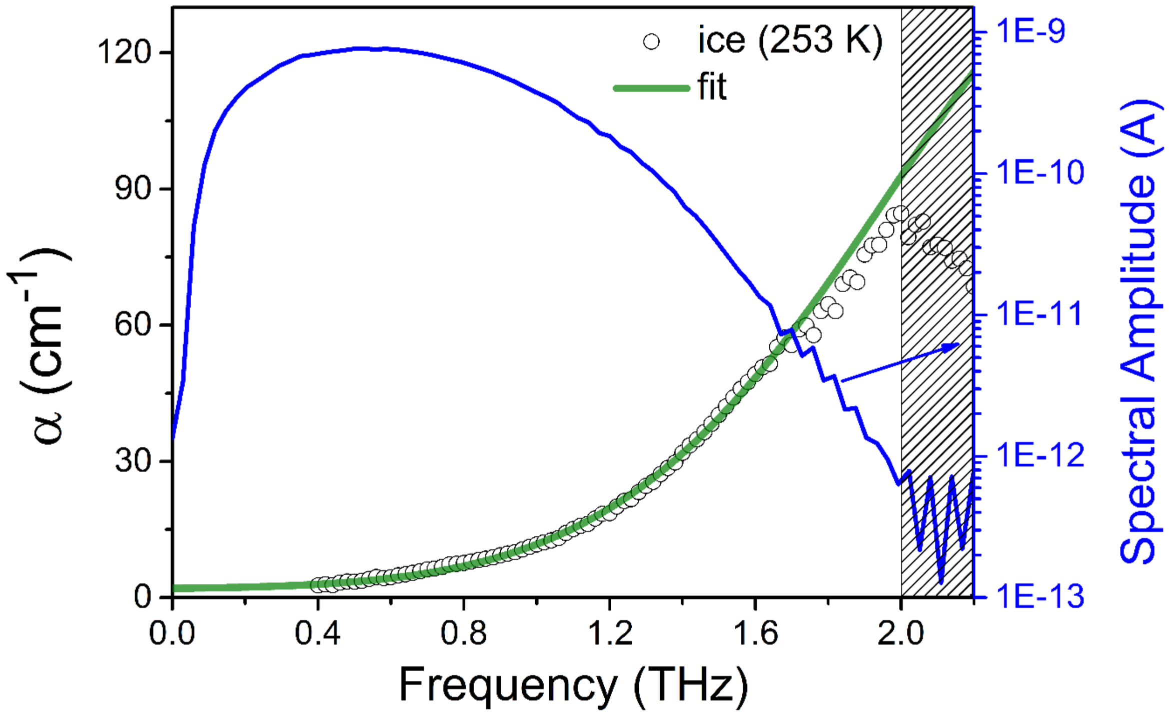

Supplementary Figure 2 | Absorption coefficient of ice (253 K) in the range of 0.4–2.0 THz. The THz

spectrum (blue line) was obtained up to 2.0 THz due to the attenuation of ice. The black open circles are the measured data for the absorption coefficient of ice. The fit (green line) shows that a Gaussian function gives good agreement with the measurement result.

Quantification of the DNA methylation signal.

A commercial biological quantification method for global DNA methylation was used to validate our THz quantification technique. The quantification kit was the Methylamp Global DNA Methylation Quantification Ultra Kit (ELISA-like reaction, Epigenek Inc.). The degree of global DNA methylation is shown in Supplementary Table 1.

Supplementary Table 1 | Relative quantification of global methylation for DNA samples using an ELISA-like method

	OD[a] average	Methylation %	Standard Deviation	Relative p value
**Samples**				
293T	0.269[1], 0.184[2]	10.744	0.006	0.421
Methylated 293T	0.628[1]	25.522	0.101	1.000
PC3	0.334[2]	23.030	0.104	0.902
A431	0.482[1]	19.533	0.024	0.765
A549	0.292[2]	19.476	0.083	0.763
MCF-7	0.386[1]	15.581	0.003	0.611
SNU-1	0.177[2]	13.004	0.046	0.510
**Control**				
Positive Control	0.600[1], 0.350[2]			
Negative Control	0.008[1], 0.060[2]			

$$\text{Methylation \%} = \frac{(\text{sample OD}^{a} - \text{Negative OD}) \,/\, \text{X}^{*}}{(\text{positive control OD} - \text{Negative control OD}) \times 10} \times 100$$

X^{*}: CG^{b} content (human DNA: 41%)

#[1, 2.] : matched controls for each sample

[a]OD: Optical Density

[b]CG: cytosine nucleotide – guanine nucleotide in the linear sequence of bases
